# Estimation of Levels of Salivary Pyridinoline Cross-Linked Carboxyterminal Telopeptide of Type I Collagen (ICTP) in Periodontally Healthy and Diseased Patients at Various Time Intervals Before and After Periodontal Therapy

**DOI:** 10.7759/cureus.66236

**Published:** 2024-08-05

**Authors:** Priyal Agrawal, Aashish Pandit, Sachin K Malagi, Dennis V Abraham, Balasubramanyam Vasant, Saurabh Tembhurne

**Affiliations:** 1 Periodontology, Maitri College of Dentistry and Research Centre, Durg, IND; 2 Periodontology, Indraprastha Dental College, Ghaziabad, IND; 3 Periodontics, Maitri College of Dentistry and Research Centre, Durg, IND; 4 Public Health Dentistry, Maitri College of Dentistry and Research Centre, Durg, IND

**Keywords:** periodontal, collagen, salivary, biomarkers, ictp

## Abstract

Introduction: The bacterial plaque in the tooth-supporting tissues is the main cause of inflammatory conditions called periodontal diseases. Thus, the aim of this study is to determine the levels of intercellular matrix protein (ICTP) in patients with gingivitis and periodontitis and those who are periodontally healthy, both before and after treatment at different times.

Materials and methods: Thirty clinical parameters (bleeding on probing, probing pocket depth, and clinical attachment loss) were measured at baseline, one month, three months, and six months after the patients were divided into three groups of 60.

Results: There was a significant difference between the two groups at all time intervals; the difference at one month was 34.77 (p=0.000). At three months, the difference became 31.25 (p=0.000) which increased to 36.62 (p=0.000) at six months.

Conclusion: When periodontal deterioration occurs, ICTP levels are higher, and when they are reduced, periodontal health is demonstrated.

## Introduction

A collection of inflammatory conditions known as periodontal diseases is primarily brought on by the accumulation of bacterial plaque in the tissues that surround teeth [1]. The mildest form of gingivitis is characterized by reversible gingival inflammation; however if not treated, it can lead to periodontitis, which is characterized by permanent loss of the alveolar bone and periodontal ligament [2]. Chapple et al. stated that smoking, genetic susceptibility, inadequate dental care, and systemic illnesses like diabetes are common risk factors [3]. If periodontal disease is left untreated, it can affect systemic health as well as functions of the oral cavity by causing tooth mobility and eventual loss of teeth [4]. Maintenance of periodontal health and halting the disease progression require early diagnosis and efficient treatment.

Periodontal biomarkers are very important for assessing the activity of disease and their treatment outcomes. Biomarkers like matrix Metalloproteinase-8 (MMP-8) and Interleukin-1β (IL-1β) contemplate inflammation and destruction of tissues in periodontitis. these biomarkers are monitored which helps in personalizing the treatment strategies and to monitor the progression of the disease [5]. Pyridinoline cross-linked carboxyterminal telopeptide of type I collagen (ICTP) is a biomarker used to assess bone resorption activity in periodontitis. Enhanced levels of ICTP indicate an increase in collagen breakdown and bone turnover rate, which are characteristic of active periodontal disease [6]. Monitoring ICTP levels provides valuable insights into the severity of disease and response to treatment, aiding in the management of periodontitis [7]. This biomarker serves as a useful predicting tool in the progression of periodontal tissue destruction and guiding therapeutic strategies. Pyridinoline cross-links are a potentially useful diagnostic tool in periodontitis because they are specific for bone resorption. This is because biochemical markers particular for bone deterioration can help distinguish between inflammation of the gingival area and present periodontal or peri-implant destruction of bone [8]. ICTP levels were also strongly correlated with whole-subject levels of several periodontal pathogens including Tanerellafor sythensis, Porphyromonas gingivalis, Prevotella intermedia, and Treponema denticola. Thus far, research evaluating gingival crevicular fluid (GCF) ICTP levels as a diagnostic indicator of periodontal disease activity has yielded encouraging findings. It has been demonstrated that ICTP significantly decreases following periodontal therapy, is an excellent indicator of future alveolar bone and attachment loss and has a strong correlation with clinical characteristics and putative periodontal pathogens [9].

Since ICTP is a specific bone biomarker, the levels will differ in patients with gingivitis to that of periodontitis and healthy individuals. Also, periodontal therapy will also affect the values of this marker in the saliva and is indicative of bone resorptive activity. Thus, the aim of the present study is to estimate the levels of ICTP in periodontally healthy individuals and compare it with their levels in patients with gingivitis and periodontitis, pre- and post-treatment at different time intervals.

## Materials and methods

The selection of patients is based on the 2017 classification of periodontal diseases. The inclusion criteria were patients in the age range of 20-50 years, patients who are willing to get enrolled in the procedure with consent, patients who have no history of any periodontal treatment and or any antimicrobial therapy for the past six months, and patients who require surgical therapy after Phase I therapy as per clinical and radiographic assessment. The following were excluded: pregnant women and lactating mothers, patients with any history of systemic disease, and the presence of any preexisting oral infective condition other than gingivitis/periodontitis.

The study was approved by the Institutional Ethical Review Board, Maitri College of Dentistry and Research Centre, Anjora, Durg, Chhattisgarh. Written informed consent was obtained, and 60 patients from the outpatient department of periodontology, Maitri College of Dentistry and Research Centre were recruited for the study as per the selection criteria.

The total sample size was 60 of three groups (each group consisted of 20 each). GROUP I is periodontally healthy individuals satisfying the ‘clinical gingival health on intact periodontium’ criteria of the 2017 classification. GROUP II is patients of ‘Gingivitis associated with dental biofilm’ as per 2017 classification. Selection of these patients is chosen on the following criteria (probing depth ≤ 3mm, no clinical attachment loss, but presence of bleeding on probing (BOP) on at least six sites, no radiographic evidence of bone loss). GROUP III is periodontitis patients (Stage III or IV of 2017 classification). Selection of these patients is done on the following criteria (probing depth > 6mm, clinical attachment level ≥ 3mm, BOP on at least six sites, radiographic evidence of bone loss in more than 30% of teeth).

Clinical parameters (BOP, probing pocket depth (PPD), and clinical attachment loss (CAL)) were measured at baseline, one month, three months, and six months. Patients in Group II and Group III received scaling combined with periodontal flap therapy. After treatment, evaluations occurred at one, three, and six months, comparing ICTP levels. Healthy patients were evaluated only at baseline.

Saliva collection procedure

**Figure 1:**
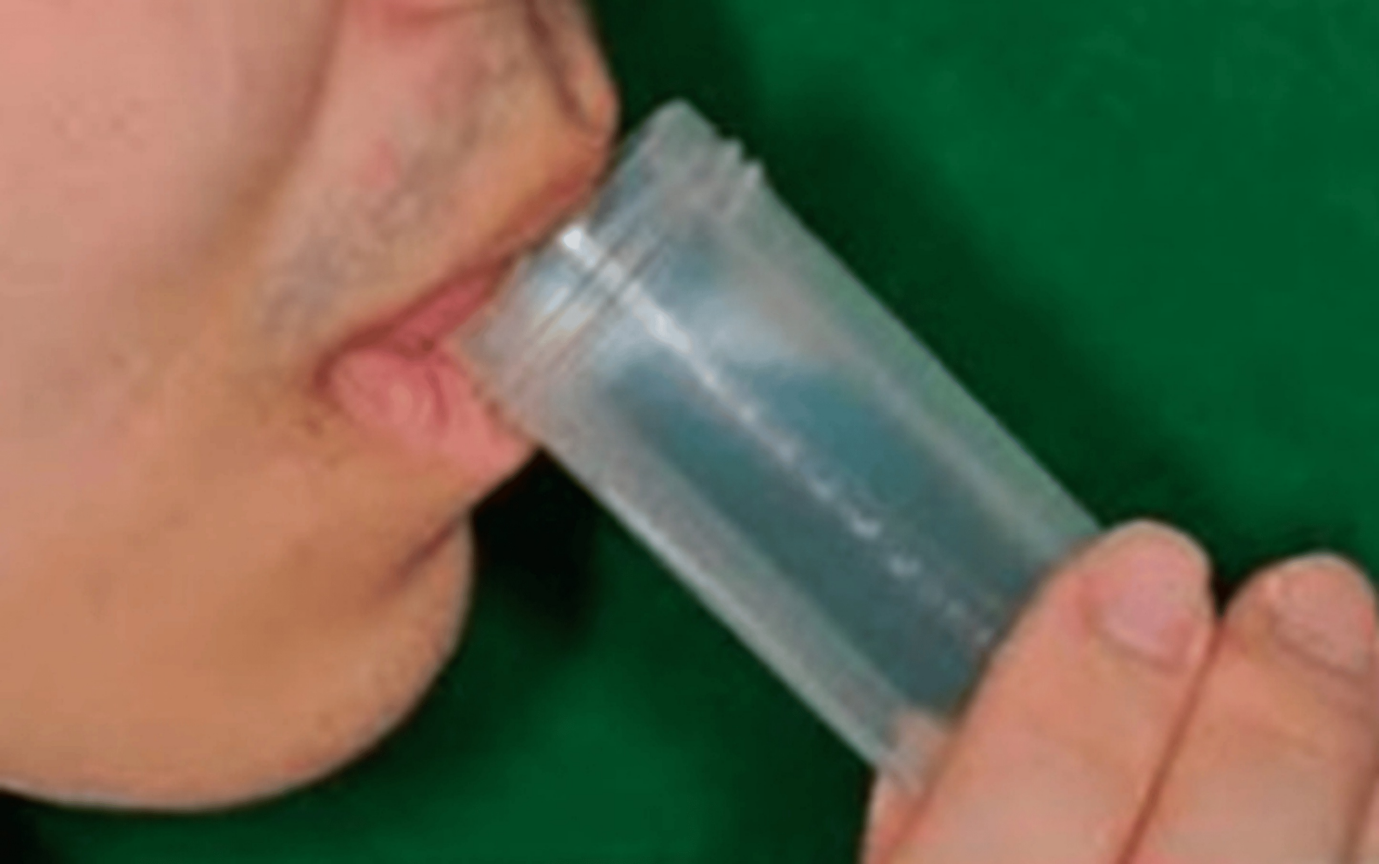
Saliva sample collection.

Saliva was collected using sterile micropipettes [10]. The samples were collected in the morning hours before breakfast and patients were advised to refrain from eating or drinking one hour before sampling. Then the subjects were asked to rinse their mouth using deionized distilled water before collection. The saliva collected was completely unstimulated whole saliva. In order to drain saliva into the sterile container while keeping their eyes open, patients were instructed to tilt their heads forward and lean over them (Figure 1). Five minutes were spent in total collecting the saliva, yielding a volume of five milliliters. Then the collected samples were transported to the site of storage encased in a box of dry ice.

Saliva processing

Samples were centrifuged at 2800 rpm and stored at -80°C until the final testing. The supernatant was removed taking care not to disturb the ‘pellet’ at the bottom and was transferred to the fractions in labeled cryotubes which were ready for storage.

Testing of samples

Samples were tested with a special ELISA kit for human ICTP (Wuhan Fine Biotech Co., Ltd.) which is based on a double-antibody sandwich technique to detect human ICTP. Complete guidelines given by the manufacturer were followed to carry out the testing once all the samples were collected and processed.

## Results

The results suggest that the level of ICTP is higher in the periodontitis group than in the gingivitis and healthy group. Its level was reduced in periodontitis and gingivitis groups after treatment.

Table 1 demonstrates the difference in mean between the groups with periodontitis and those in good health is 47.72 (p<0.001). A similar finding was seen when the gingivitis and periodontitis group was compared at baseline with a mean difference of 42.02 (p<0.001). However, the difference between healthy and gingivitis was not significant with a mean difference of 5.70 (p=0.055).

**Table 1:** Post hoc Tukey test showing comparison of baseline values among the three groups. * means statistically significant

(I) group	(J) group	Mean Difference (I-J)	Std. Error	Sig.	
					Healthy	Gingivitis	-5.7	2.406	0.054	
Periodontitis	-47.72^*^	2.406	<0.001*		
Gingivitis	Healthy	5.7	2.406	0.054	
	Periodontitis	-42.02^*^	2.406	<0.001*	
Periodontitis	Healthy	47.72^*^	2.406	<0.001*	
	Gingivitis	42.02200^*^	2.406	<0.001*	

Table 2 shows the comparison of gingivitis and periodontitis patients at follow-up intervals at one month, three months, and six months. An independent t-test was employed. Between the two groups, there was a difference of statistical significance at every time point; the variation at one month was 34.77 (p<0.001). At three months, the difference became 31.25 (p<0.001) which increased to at six months 36.62 (p<0.001).

**Table 2:** Independent t-test showing comparison of values at various follow-ups among gingivitis and periodontitis groups. * means statistically significant

	Group	N	Mean	Std. Deviation	Std. Error Mean	T value	P-value (sig)
One month	Gingivitis	20	11.82	3.82	0.854	-11.744	<0.001*
	Periodontitis	20	46.59	12.67	2.834		
Three months	Gingivitis	20	8.62	2.75	0.616	-12.564	<0.001*
	Periodontitis	20	39.87	10.77	2.41		
Six months	Gingivitis	20	8.18	2.91	0.651	-11.593	<0.001*
	Periodontitis	20	44.8	13.85	3.097		

Table 3 shows that there was a significant difference from baseline to one month, baseline to three months, and baseline to six months (p<0.001). The difference at one month from baseline was 9.05, at three months from baseline was 12.25 and at six months from baseline was 12.69.

**Table 3:** Within-group comparison using the paired t-test at various follow-ups as compared to the baseline of gingivitis patients. * means statistically significant

	Mean	Std. Deviation	Std. Error Mean	t	df	Sig. (2-tailed)
Baseline – one month	9.05	4.28	0.958	9.444	19	<0.001*
Baseline – three months	12.25	4.69	1.05	11.67	19	<0.001*
Baseline – six months	12.69	4.72	1.055	12.028	19	<0.001*

Table 4 shows a significant difference from baseline to one month, baseline to three months, and baseline to six months (p<0.001). The difference at one month from baseline was 16.30, at three months from baseline was 23.02, and six months from baseline was 18.02.

**Table 4:** Within-group comparison using the paired t-test at various follow-ups as compared to the baseline of periodontitis patients. * means statistically significant

	Mean	Std. Deviation	Std. Error Mean	t	df	Sig. (2-tailed)
Baseline – one month	16.3	13.36	2.988	5.457	19	<0.001*
Baseline – three months	23.02	8.75	1.957	11.765	19	<0.001*
Baseline – six months	18.02	11.9	2.662	6.769	19	<0.001*

## Discussion

To shed light on and forecast the course of the disease in the future, a variety of turnover and biochemical markers found in oral fluids may be helpful. Bone turnover marker levels that are circulating may be able to shed light on the systemic signs of periodontal tissue degradation [11]. As a result of degradation of collagen and resorption of alveolar bone, ICTP is released into the periodontist tissues [12]. Research evaluating the function of intercellular matrix protein concentrations in GCF or peri-implant crevicular fluid as an indicator for diagnosis of periodontal destruction activity has yielded encouraging findings thus far. It was proposed that ICTP could be reduced after periodontal therapy, estimating further bone loss, and correlate with clinical parameters and potential pathogens of periodontal disease [13].

It has been demonstrated that saliva has great potential as a tool for tracking systemic and oral diseases and for early diagnosis. Whole saliva most closely mimics the predominate intraoral condition because it contains GCF, immune cells, and tissue metabolites. In comparison to the results obtained when using gum, citric acid, or paraffin wax, the degree of stimulation was minimal when we collected and examined whole saliva [14]. The study compared 60 patients comprising equal patients in three groups which were healthy, gingivitis, and periodontitis groups. The results of the present study show that healthy patients had least ICTP values in their saliva which were more in gingivitis patients but maximum in periodontitis patients. The mean value obtained from healthy patients was 15.17±4.90. The gingivitis group showed a mean value of 20.88±5.88 while the periodontitis group showed a mean of 62.90±10.72. The results obtained were similar to those of Palys et al. [15] and Mishra et al. [16]. Ozcaka et al. have shown contrasting results where ICTP values show no significant result from healthy to periodontitis patients [17].

When the three groups were compared, there was a difference obtained between the healthy and periodontitis groups as well as the gingivitis group and periodontitis group (p<0.001) which was significant statistically. There was no statistically significant difference between the healthy and gingivitis groups. A possible explanation could be that ICTP is a specific bone destruction bone marker. Thus, periodontitis with clinical and radiographical signs of alveolar bone loss will have a higher ICTP value as it would be circulating in serum as well as saliva. The samples of gingivitis were obtained one month, three months, and six months after the non-surgical therapy rendered to the patients, periodontitis patients were also assessed at the same time interval after non-surgical as well as surgical therapy. There was a steady decline in both the groups of ICTP values from baseline to follow-up intervals. In gingivitis, the mean value obtained at baseline was 20.88±5.88 to 8.18±2.91 at six months with a mean difference of 12.69. The difference from baseline to each follow-up interval was statistically significant (p<0.001). A similar pattern was observed in the periodontitis group where the mean value of 62.90±10.72 at baseline was reduced to 44.88±13.85 at six months. The difference was also statistically significant (p<0.001) for each interval when compared to baseline with a mean reduction of 18.02. This was similar to that of Mishra et al. [16] and Oringer et al. [13], where they concluded that the level of ICTP can assess the periodontal disease progression. Kinney et al. [18] and Gursoy et al. [19] found in their investigation that periodontitis samples had higher salivary ICTP concentrations than healthy samples. They also concluded that an increase in ICTP levels could be used as a predictor of diseased periodontal status.

These products are found in greater concentrations in the blood as periodontal disease becomes more severe. ICTP is therefore regarded as a particular biomarker of periodontal tissue degradation [20]. ICTP, or pyridine cross-linked carboxy-terminal telopeptide of type I collagen, is the predominant form of type I collagen found in alveolar bone. In progressive periodontal disease, bacterial collagenase or pro-inflammatory mediators degrade and release intercellular calcium transport protein [8]. Consequently, ICTP is thought to be unique to alveolar bone loss and is therefore particularly relevant for the prompt diagnosis of periodontitis. In their study, Giannobile et al. also came to the conclusion that pyridinoline cross-links are active markers of periodontitis-related alveolar bone loss and that their presence can be used to distinguish periodontitis from active gingivitis. The current study supported the findings of the earlier research, showing that patients with periodontitis had higher concentrations of ICTP, a breakdown product of type I collagen found in alveolar bone. Patients who were in good periodontal health had the lowest concentration [8].

## Conclusions

Considering certain limitations, the relation of ICTP with environmental factors such as smoking has not been assessed. Also, it is tedious, and long processing is required for quantification of samples. Within the limitation of this study, it can be concluded that higher values of ICTP are present in the case of periodontal destruction and its reduction shows periodontal health. Thus, it can be used as a biomarker effectively for assessing the progression of the disease and the overall state of the periodontal tissues. To determine a relationship between the ICTP values and specific clinical parameters, more research with a larger sample size is required.
